# A Rare Case of Thromboangiitis Obliterans of Bilateral Upper Extremities in an Adult Male

**DOI:** 10.7759/cureus.24975

**Published:** 2022-05-13

**Authors:** Toluwalope F Ejiyooye, Abimbola O Ajibowo, Sudha Dirisanala, Bukola Olagbende, Uchenna E Ezenagu, Aadil Khan

**Affiliations:** 1 Family Medicine, Brooke Army Medical Center, San Antonio, USA; 2 Internal Medicine, Lugansk Medical University, Luhansk, UKR; 3 Internal Medicine, Konaseema Institute of Medical Sciences and Research Foundation, Amalapuram, IND; 4 Internal Medicine, Meharry Medical College, Nashville, USA; 5 Medicine, Sumy State University, Sumy, UKR; 6 Internal Medicine, Lala Lajpat Rai (LLR) Hospital, Kanpur, IND

**Keywords:** ischemia, amputation, pharmacotherapy, beurger disease, thromboangiitis obliterans

## Abstract

Thromboangiitis obliterans (TAO) also known as Buerger’s disease is a nonatherosclerotic vasculitis that is more common in adult male smokers. TAO predominantly affects lower extremities, however, cases with bilateral upper extremities involvement are a rare entity and difficult to manage. Symptoms of the disease mostly mimic that of acute ischemia of the limb. Diagnosis is mostly made clinically, however, radiographic vascular evaluation can strengthen the diagnosis. Herein we present a case of a 41-years-old male with a history of chronic smoking whose signs and symptoms were suggestive of TAO in bilateral upper extremities, however, the patient did not respond to smoking cessation and pharmacotherapy for a long time owing to which he underwent amputation after taking proper informed consent.

## Introduction

Thromboangiitis obliterans (TAO) is a nonatherosclerotic, highly inflammatory, segmental vascular occlusive pathological entity, that predominantly affects small and medium-sized distal arteries and veins and is aggravated by substantial tobacco exposure [1]. Incidences of TAO in the lower extremity are much more common, however, cases with bilateral upper limb involvement are a very rare entity and challenging to treat [2]. Even though TAO commonly involves the extremities, certain cases have shown that the disease process can affect other sites such as major vessels like aorta, coronary and mesenteric arteries [3]. Although etiopathogenesis is debatable, multiple risk factors like chronic smoking, narcotics, and tobacco intake can lead to TAO. Studies have shown that autoimmunity, hereditary, and immunological factors are contributing to the pathogenesis of TAO [4]. Thromboangiitis obliterans (TAO) also known as Buerger's disease (BD) can have a myriad of clinical manifestations such as Raynaud's phenomenon, ischemic ulcers, gangrene in the extremities, and superficial vein thrombosis [5,6]. Diagnosis is confirmed by clinical and vascular evaluation, including age (<50 years), history of smoking and/or tobacco intake, and distal extremity ischemia. Angiographic findings consistent with TAO are important criteria to make a diagnosis according to Olin, Shionoya, and Papa et al. [5,7,8]. The conventional treatment is complete abstinence from smoking, however, the use of aspirin, Iloprost, and streptokinase are recommended. Sympathectomy and vascular bypass procedures are some of the surgical approaches. Amputation is a last resort if there is no improvement to conventional and other treatments [9]. Herein, we present a case of a 41-years-old male who has thromboangiitis obliterans in advanced stage involving bilateral upper limb extremities. The IRB approval was taken from Ganesh Shankar Vidyarthi Memorial Medical College Ethics Committee (approval number: EC/BMHR/2022/47).

## Case presentation

We discuss a case of a 41-year-old adult male who presented to the surgery department with complaints of black discoloration of hand in bilateral upper extremities associated with burning pain and tingling sensation for the past four months which first started in the left hand and then progressed to the right hand. He had been seeking treatment from a local practitioner but he had partial relief. The patient is a known diabetic and had a history of smoking of 20 pack-year for the last 15 years and still consumes tobacco. The pain was acute in onset, radiating to arms, and relieved by movement.

**Figure 1:**
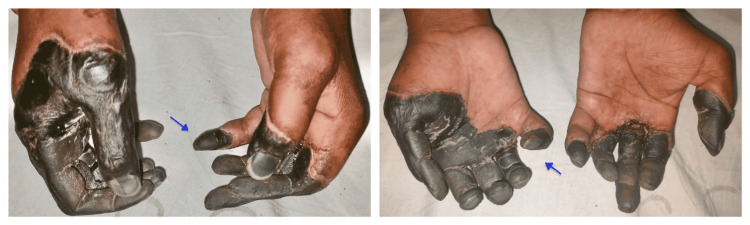
Dry gangrenous changes in both hands.

Clinical examination revealed dry gangrenous changes over digits of bilateral upper extremities extending from the metacarpophalangeal (MCP) joint to the tip of fingers with thinning out of distal phalanx and atrophy of the nails (Figure 1). In the uninvolved area, skin appeared erythematous with diffusely edematous up to the metacarpophalangeal (MCP) joint. Brachial pulses were present on both sides, however, right ulnar pulse was feeble, while the ulnar pulse was absent on the left side. The lower limb examination was unremarkable.

**Figure 2:**
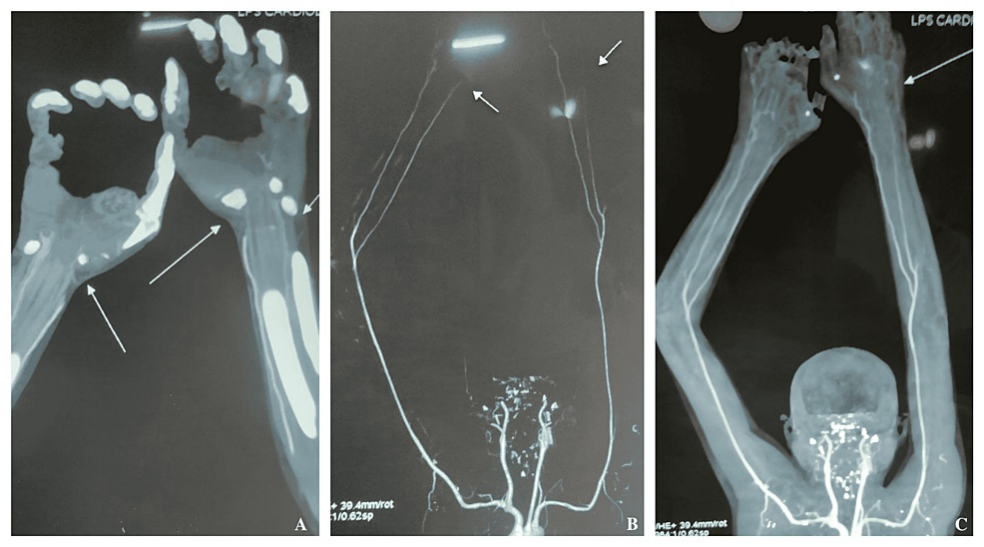
CT angiography showing bilateral poor opacification of palmar and digital arteries (A) and stenosis of left and right distal ulnar artery (B and C).

Neurological examination of extremities revealed hand muscle weakness with grade 0/5 power and loss of sensation on the gangrenous area while in the rest of the hand sensation was intact. However, the nerve conduction study was normal. His blood investigations were within normal limits. Examination of the remaining systems was unremarkable. On coupling his signs and symptoms the diagnosis of upper limb ischemia due to TAO was made. Further assessment using CT-angiography showed occlusion of ulnar arteries bilaterally with uneven opacification of palmar and digital arteries (Figures 2A-2C).

After consultation with the vascular team program at our hospital operative partial amputation of involved digits was recommended. Informed consent was taken and the patient prepared for surgery. The surgery went successful without any complications. He was advised to stop tobacco intake immediately and was treated with nifedipine 20 mg twice a day, aspirin 75 mg once a day, and atorvastatin 20 mg twice a day. On follow-up, after two weeks patient responded to treatment and his condition improved. Patient consent was taken before writing this case report.

## Discussion

Thromboangiitis obliterans (TAO) is most prevalent in males aged 40 to 50 years as in our case and less common in women [10,11]. Thromboangiitis obliterans is characterized by a hyper immunological response that produces a cellular intraluminal thrombus sparing the vessel wall. While understanding of pathogenesis is still very narrow but delayed-type hypersensitivity to collagen secondary to cigarette smoking (termed “toxic angiitis”) antiendothelial antibodies formation, and hypercoagulable disorders (prothrombin gene mutation 20210 and anticardiolipin antibodies) are some of the proposed complementary factors [12-15].

Classical signs and symptoms which are suggestive of thromboangiitis obliterans (TAO) are due to ischemia of affected limb and these are persistent pain or claudication of affected extremities, pale appearance, perishingly coldness, loss of pulse, and abnormal sensation in limbs moreover in advanced stage dry gangrene and paralysis of extremities are hallmark clinical manifestation of TAO. In our case, the patient developed dry gangrenous changes over digits of bilateral upper extremities associated with loss of motor and sensory functions.

Indeed, the addition of histopathological examination showing acute phase inflammatory cell thrombus and imaging techniques such as color-duplex ultrasound (US), magnetic resonance angiography (MRA), and computed tomography angiography (CTA) would significantly strengthen the definitive diagnosis. However, clinical and radiological vascular evaluation remains the gold standard criteria to diagnose TAO. The most acceptable are Shionoya and Olin criteria [7,15]. Components of this criteria include a history of smoking in men up to age 50 years with or without thrombophlebitis and involvement of extremities without diabetes, hyperlipidemia, or thrombo-embolic features are required for making a diagnosis of TAO. In our case, the patient reported having a history of smoking for more than 15 years and the involvement of bilateral upper extremities.

The mainstay treatment for TAO is conservative and smoking cessation remains the utmost priority despite various other medical and surgical treatments being proposed. Research has shown that smoking cessation can significantly prevent disease progression and can lead to symptomatic improvement of lesions and for which various pharmacotherapy (bupropion or varenicline) and group therapy are recommended [16]. The pharmaceutical treatment is mainly focused on improving peripheral blood flow in affected limbs, however, reversing of disease symptoms can be seen but the long-term prognosis is still very limited especially if the disease progress to an advanced stage. Vasodilators and antiplatelet drugs are useful however, not enough evidence found to recommend them as the first-line treatments. Surgically vascular bypass surgery and sympathectomy can help improve pain and heal ulcers.

Finally, amputation is the last choice if irreversible ischemia and dry gangrene develops as it was found in our case where the patient underwent amputation of the affected extremity after proper written consent. Recent advances show that VEGF (vascular endothelial growth factor) and autologous bone marrow mononuclear cell implantation can be helpful, but it is yet to get final approval.

## Conclusions

Thromboangiitis obliterans (TAO) affecting bilateral upper extremity is a very rare entity and despite various therapeutic interventions disease remains challenging to treat. As in our cases patients presented to us with dry gangrene and paralysis in bilateral upper limbs for which the patient underwent amputation. The patient responded well to treatment and was kept under follow-up. Further research is needed for evolving therapeutics to improve quality and better disease management.
